# Stigmatizing attitudes towards mental illness among university students: a comparative study with the general population

**DOI:** 10.47626/2237-6089-2023-0708

**Published:** 2024-10-28

**Authors:** Beatriz Atienza-Carbonell, Vicent Balanzá-Martínez, Alberto Bermejo-Franco, Laura Carrascosa-Iranzo

**Affiliations:** 1 Valencian International University Valencia Spain Valencian International University (VIU), Valencia, Spain.; 2 Universitat de València VALencia Salut Mental i Estigma (VALSME) Research Group Valencia Spain VALencia Salut Mental i Estigma (VALSME) Research Group, Universitat de València, Valencia, Spain.; 3 Consorcio Hospital General Universitario de Valencia Clinical Documentation and Admission Unit Valencia Spain Clinical Documentation and Admission Unit, Consorcio Hospital General Universitario de Valencia, Valencia, Spain.; 4 Universitat de València Teaching Unit of Psychiatry and Psychological Medicine Department of Medicine Valencia Spain Teaching Unit of Psychiatry and Psychological Medicine, Department of Medicine, Universitat de València, Valencia, Spain.; 5 Instituto de Salud Carlos Centro de Investigación Biomédica en Red de Salud Mental Madrid Spain Centro de Investigación Biomédica en Red de Salud Mental (CIBERSAM), Instituto de Salud Carlos III (ISCIII), Madrid, Spain.; 6 Universidad Europea de Madrid Faculty of Sports Sciences Madrid Spain Faculty of Sports Sciences, Universidad Europea de Madrid, Madrid, Spain.

**Keywords:** Social stigma, attitudes, mental disorders, university students

## Abstract

**Objective:**

The aim of this study is to compare stigmatizing attitudes, reported and intended behavior, and knowledge of mental illness between university students and the general population.

**Methods:**

An online cross-sectional observational study was conducted. The survey included sociodemographic data and validated stigma questionnaires (the Attribution Questionnaire [AQ-27], the Reported and Intended Behaviour Scale [RIBS], and the Mental Health Knowledge Schedule [MAKS]). Descriptive statistics, bivariate analyses, and multiple regression modeling were employed to analyze the data.

**Results:**

A total of 506 participants completed the survey, including 226 (44.7%) university students (61.1% women), and 280 (55.3%) individuals from the general population (69.3% women). For both groups, women and individuals who had lived with someone with mental health problems exhibited more positive attitudes (p < 0.05). University students reported greater knowledge of mental illness (p < 0.05) than the general population. After controlling for covariates, university students only scored higher than the general population in the blame factor of the AQ-27 (p < 0.05). Additionally, older participants from both groups exhibited higher levels of stigmatizing attitudes compared to those of a younger age.

**Conclusion:**

These findings suggest that university students exhibit similar levels of stigmatizing attitudes to the general population. Among both groups, female sex, older age, previous contact with individuals with mental illness, and greater knowledge of mental health are all associated with less stigma toward people with mental illness. Tailored interventions grounded in contact with mental illness have the potential to help reduce stigmatizing attitudes within both groups.

## Introduction

From a social perspective, stigma refers to adopting discriminatory behaviors, prejudiced attitudes, negative emotional responses, and biased social structures towards members of a subgroup of society.^1^ The literature shows that individual, interpersonal, and structural stigma associated with mental illness is prevalent worldwide.^2^

According to Corrigan et al.,^3^ social stigma comprises three components: stereotypes, prejudice, and discrimination. Stereotypes are knowledge structures that most members learn of a social group. Prejudice supports negative stereotypes and generates adverse emotional reactions as a result. Prejudice leads to discrimination, that is, behavioral reactions. As a result, individuals with mental illness are less likely to be employed, rent accommodation, or have social interactions and are more likely to be falsely charged with crimes than the general population.^3,4^

Discrimination of individuals with mental illness in the workplace due to stigmatizing attitudes is a prevalent problem across different countries and cultures.^5^ A study published by the Organization for Economic Co-operation and Development (OECD) shows that individuals with a common mental illness (e.g., anxiety or depression) are three times more likely to be unemployed.^6^ This probability increases up to seven times more in individuals with a severe mental illness (SMI) (e.g., schizophrenia or bipolar disorder).

Notably, labeling mental disorders has been shown to have a different impact depending on the type of mental illness. Labeling a person with schizophrenia has a negative effect on social attitudes, whereas depression has been shown to have no substantial impact on social attitudes.^7^ This is likely because depression is more accepted by the general population than SMIs such as schizophrenia.^2^ Additionally, among the general population, men have been observed to have significantly higher levels of stigmatizing attitudes towards mental illness than women.^7,8^

To date, numerous studies have been conducted in several countries to analyze social stigma towards individuals with mental illness in specific populations, such as individuals with mental health problems (internalized stigma or self-stigma),^9^ healthcare professionals,^10^ and university students, especially those in health sciences.^11–15^ Studies conducted among university students have observed a range of attitudes toward mental illness, including positive and negative perceptions.^14,15^ A recent study observed that female university students showed less stigmatizing attitudes than male students, but similar stereotypes and prejudice toward people with mental disorders.^11^

Several studies support the theory that increased education and exposure to mental illness, e.g., previous contact with people with mental illness, are associated with reduced stigma.^13,16–18^ For instance, it was observed that psychiatrists, who possess a higher level of familiarity with mental illness, exhibited significantly lower levels of stigmatizing attitudes compared to undergraduate students and medical students.^18^ Another study found that older college students and individuals who were more familiar with mental illness were less likely to stigmatize and maintain social distance from people with mental illness.^17^ Additionally, college students who believed that personality traits were unchangeable demonstrated a higher tendency to stigmatize people with mental disorders and desired more social distance from them. In contrast, previous contact with individuals experiencing a mental illness predicted a reduced desire for social distance.^13^ In sum, variables such as age, sex, educational level, and previous contact with people with mental illness may have an influence on the degree of social stigma.

The university population may be susceptible to short- and long-term educational interventions to reduce social stigma.^19^ There is also evidence that intervention programs delivered in an academic setting are more effective than large-scale information campaigns targeting the general population.^20^ Therefore, comparing the levels of social stigma among university students with those in the general population and identifying common and specific features associated with stigma across both groups would allow the design of more tailored anti-stigma interventions.^21^

Of note, research about social stigma among university students has mainly assessed this group in isolation, and very few studies contrasted the level of stigma with that of other population groups (e.g., journalists, healthcare professionals).^18,22,23^ To our knowledge, only two studies have compared university students and individuals from the general population, both conducted in Arab-Islamic countries.^24,25^ One study found similar attitudes toward people with mental illness across three groups – medical students, relatives of psychiatric patients, and the general population from Oman.^24^ Interestingly, sex and previous contact with people with mental illness had no significant effect on attitudes, whereas younger participants showed a trend to more favorable attitudes. Conversely, in Pakistan, the general public showed a significantly higher degree of stigma towards mental illness, measured as social distancing, than both healthcare students and healthcare professionals.^25^ Thus, having more knowledge about mental illness may be related to lower levels of stigma.^18,25^ Moreover, being male and being over the age of 30 years were associated with higher levels of stigmatizing attitudes in that study.^25^ To our knowledge, no similar comparative study has been conducted in Western societies. In addition, the existing studies assessed one component of social stigma only and recruited a subset of the population of interest, e.g., medical and healthcare students.^24,25^ Thus, a more comprehensive characterization of social stigma among university students versus the general population is currently lacking.

The main objective of this study is to compare the degree of social stigma towards mental illness of university students with that of the general population from Spain. A secondary study aim is to examine the relationship between stigmatizing attitudes, reported and intended behaviors, previous contact with people with mental health problems, and variables of interest such as sex, knowledge about mental illness, and degree of familiarity/previous contact with individuals with mental illness.

Therefore, the following main hypothesis is proposed: university students will have fewer stigmatizing attitudes than the general population. Furthermore, women and those with previous contact with individuals with mental illness are expected to present a lower level of social stigma.

## Methods

### Study design, participants, and setting

This study is part of the VALencia Stigma in Medical Education (VALSME) research group, which has been evaluating the presence of stigma towards mental illness and mental health issues among university students at the University of Valencia, Spain, since 2017.^26–28^

This was an observational, cross-sectional study with a survey sample.

The inclusion criteria for the study were that the university students should be enrolled on Bachelor's, Master's, or Ph.D. degrees at any University in Spain in the academic year 2021-2022. The participants from the general population should be living in Spain during their participation in the study and should be over 18 years of age. Subjects who did not agree to participate were excluded from the study.

Considering the proportion of internet users in Spain (62% of the population, 31,872,000 citizens), assuming a heterogeneity of 50% and applying a margin of error of 5% with a confidence level of 95%, the minimum sample size was determined to be n = 385 (n = 193 in each group).

### Study procedures

During 6 weeks in May and June 2022, university students and the general population were invited to complete a self-administered online survey using LimeSurvey®. Using the snowball technique, a convenience sample was recruited through university and social networks (Facebook, LinkedIn, Twitter). This technique has been widely used in similar studies.^14,26,29–31^

### Ethical considerations

Participation was voluntary and no academic or economic compensation was offered. The participants included in the study provided written consent. The study was conducted following the ethical principles of clinical research involving humans (World Medical Association [WMA], Declaration of Helsinki). The research project was reviewed and approved by the Human Research Ethics Committee (CEIH) of the Universitat de València (UV-INV_ETICA-2022042) before the start of the study.

### Variables and instruments

All participants were invited to complete an online survey including the following sociodemographic variables: sex, age, occupational status, educational level, level of current studies, marital status, and personal history of mental disorders.

Regarding the group variable, participants who reported studying on undergraduate, Master's, or Doctoral programs when completing the survey were categorized as university students, while the remaining participants were categorized as the general population.

Additionally, participants were invited to complete the questionnaires described below.

**Attribution Questionnaire (AQ-27).** The AQ-27 is an instrument that assesses attitudes towards mental disorders.^32^ This study used the Spanish adaptation validated by Muñoz et al.^33^ The AQ-27 presents a vignette about a person with schizophrenia and subsequently includes 27 items grouped into nine factors with three questions each. The subscales correspond to blame, anger, pity, help, dangerousness, fear, avoidance, segregation, and coercion. Items are rated on a 9-point Likert-type scale, and subscale scores are calculated by summing the items corresponding to that subscale. Higher factor scores represent a greater endorsement of the corresponding attitude or belief. The Cronbach's alpha internal consistency coefficient is close to 0.855. In the present study, the sample alpha coefficient was 0.876, indicating a high internal consistency level.

**Reported and Intended Behaviour Scale (RIBS).**^34^ The RIBS is aimed to evaluate future intentionality related to the stigma towards mental illness. The RIBS scale is made up of eight items. Items 1-4 estimate the prevalence of behaviors and how participants might or might not have engaged in those behaviors. These items are not scored. According to the RIBS, previous contact with people with mental health problems is defined as having known a neighbor, having lived with, having worked with, or having had a friend with a mental illness. Items 5-8 are scored on a Likert-type scale from 1 to 5 (1 = strongly disagree; 5 = strongly agree). The scale's psychometric properties are reliable, with a Cronbach's alpha of 0.75. In the present study, the sample alpha coefficient was 0.83.

**Mental Health Knowledge Schedule (MAKS).**^35^ The 12-item MAKS assesses knowledge regarding mental illness stigma. The MAKS comprises six stigma-related mental health knowledge areas: help seeking, recognition, support, employment, treatment, and recovery, and six items that enquire about knowledge of mental illness conditions. The items are scored on a 5-point Likert scale, ranging from 1 = strongly disagree to 5 = strongly agree. Total scores are calculated by adding together the response values of each item ("Don't know" is coded as neutral = 3). Items 6, 8, and 12 are reverse coded. Overall, higher scores indicate greater knowledge. Because the MAKS scale was not designed to be used as a functional scale, Cronbach's alpha is not a determining factor of the reliability of this scale.

The self-report instruments chosen in the present study are among the most commonly used questionnaires to comprehensively assess social stigma, including knowledge, attitudes, and behaviors in university students and the general population.

### Statistical analysis

To describe the distribution of the sociodemographic characteristics of the sample, measures of central tendency (mean) and dispersion (standard deviation [SD]) were used for the quantitative variables and absolute (n) and relative frequencies (%) were used for qualitative variables.

Associations between categorical variables were measured using the Pearson chi-square test (χ^2^). We operationalized previous contact with individuals experiencing mental health problems in four categories: knowing a neighbor with a mental illness, living with someone with a mental illness, working with individuals who have mental health issues, or having a friend with a mental illness. To investigate the relationships between the two distinct groups, university students and the general population, in relation to the aforementioned variables, we conducted statistical analysis using Pearson's χ^2^.

Parametric tests were used, taking into account that the sample size (n > 30) assumed the normality of the sample (central limit theorem). Levene's test was run to study the homogeneity of variances of the sample. Continuous variables were compared using Student's *t* test for two independent groups (e.g., sex, diagnosis of mental illness) or one-way analysis of variance (ANOVA) in the case of three or more independent groups (e.g., educational level, current studies). If the main effect was significant, pair-wise comparisons were made using the Bonferroni post hoc test to compare parametric variables of more than two groups. Effect sizes were calculated using Cohen's *d* and eta squared (η²). Additionally, we conducted a stratified analysis by sex to explore potential sex-specific associations within the two study groups. Due to a very low sample size in certain categories of the marital status variable, we redefined marital status into two variables: single (encompassing those who were single, separated or divorced, or widowed) and in a couple (encompassing those who were in a couple or married).

The influence of individual variables on AQ-27 scores was examined through multiple linear regression modeling. The factors of the AQ-27 were analyzed as dependent variables separately. Linear regression assumptions, including linearity, homoscedasticity, and normality and independence of residuals, were assessed graphically. Group (university students or general population), age, and MAKS total scores were defined *a priori* as independent variables and were entered in a single step.

Statistical analysis was performed with the IBM SPSS program (version 26), considering that a relationship is statistically significant when p > 0.05.

## Results

### Sample description

The sample comprised 506 participants – 226 (44.7%) university students and 280 (55.3%) individuals from the general population.

Table 1 describes the sociodemographic variables of both groups. In the general population, the mean age was 42.12 years (SD = 13.31), while university students were a mean of 31.49 years old (SD = 10.74, t [504] = 9.939, p < 0.001). There were no differences between the two groups in terms of sex (69.3% of women in the general population and 61.1% in university students) (χ^2^ [2, n = 506] = 3.749, p = 0.153). There were also no differences between the groups regarding a previous diagnosis of any mental illness, with approximately 15% in both cases (χ^2^ [1, n = 506] = 0.009, p = 0.922).

**Table 1:** Sociodemographic characteristics among university students and the general population

Variables		University students (n = 226) Mean (SD)	General population (n = 280) Mean (SD)	*t* test	p-value
Age		31.49 (10.74)	42.12 (13.31)	9.939	< 0.001
		**n (%)**	**n (%)**	**x** ^2^	
Sex					
	Non-binary	1 (0.4)	1 (0.4)	3.749	0.153
	Female	138 (61.1)	194 (69.3)		
	Male	87 (38.5)	85 (30.4)		
					
Educational level					
	Secondary education or lower	0 (0.0)	26 (9.8)	-	-
	Vocational training	0 (0.0)	50 (18.9)		
	University degree	0 (0.0)	188 (71.2)		
					
Current studies					
	Secondary education	0 (0.0)	3 (1.1)	506	< 0.001
	Vocational training	0 (0.0)	13 (4.6)		
	University degree	93 (41.2)	0 (0.0)		
	Master's degree	119 (52.7)	0 (0.0)		
	Doctorate	14 (6.2)	0 (0.0)		
					
Marital status					
	Single	92 (40.7)	60 (21.4)	39.383	< 0.001
	In a couple	74 (32.7)	73 (26.1)		
	Married	49 (21.7)	118 (42.1)		
	Separated or divorced	11 (4.9)	25 (8.9)		
	Widowed	0 (0.0)	4 (1.4)		
					
Mental illness diagnosis					
	Yes	34 (15.0)	43 (15.4)	0.009	0.922

SD = standard deviation; x^2^ = chi-square test.

Data expressed as * mean ± standard deviation, t test for independent samples; or † absolute frequency (%), chi-square test of association.

Regarding the general population's education level, most of the participants in this group held a university degree (71.2%). Almost a fifth (18.9%) had studied vocational training, while only 9.8% held a secondary education diploma or lower.

As for the studies they were currently pursuing, most of the university students in the sample were enrolled on Master's degrees (52.7%), compared to 41.2% for Bachelor's degrees, and 6.2% for doctoral studies. In the general population, only 5.7% were studying in secondary education or vocational training (χ^2^ [5, n = 506] = 506, p < 0.001).

For the total sample (n = 506), the majority reported having had previous contact with people with a mental illness, whether they cohabited with them (37.3%), were a neighbor (39.1%) or a close friend (62.1%). The sample included 43.7% of people who had ever worked with someone with a mental health problem.

### Between-group comparison of stigmatizing attitudes, knowledge, and reported and intended behaviors

Table 2 describes the total scores and differences between groups for stigma associated with mental illness measured by the AQ-27, the RIBS, and the MAKS. Notably, only the help factor of the AQ-27 presented significant differences in the level of stigma between both groups, with a higher score among university students and a small effect size (p = 0.025; *d* = 0.20). In addition, a borderline significant higher score was also found in the general population for the coercion factor (p = 0.054; *d* = 0.174) of the AQ-27.

**Table 2:** Comparison of total scores in AQ-27, RIBS, and MAKS between university students and the general population

Variables*		University students (n = 226) Mean (SD)	General population (n = 280) Mean (SD)	*t* test	p-value	Cohen's *d*
AQ-27						
	Blame	8.63 (3.91)	8.19 (3.47)	-1.337	0.182	0.119
	Pity	17.42 (4.63)	17.44 (4.64)	0.044	0.965	0.004
	Anger	7.22 (4.06)	7.41 (3.86)	0.526	0.599	0.047
	Dangerousness	10.00 (5.54)	10.36 (5.44)	0.752	0.453	0.067
	Fear	7.82 (5.57)	8.38 (7.82)	1.141	0.254	0.102
	Help	23.09 (3.79)	22.27 (4.42)	-2.248	0.025	0.200
	Coercion	18.01 (5.48)	18.92 (4.93)	1.929	0.054	0.174
	Segregation	8.16 (4.90)	8.10 (4.77)	-0.139	0.889	0.012
	Avoidance	11.90 (5.75)	12.50 (5.99)	1.151	0.250	0.103
						
Total RIBS		15.57 (3.59)	15.09 (3.46)	-1.541	0.124	0.138
Total MAKS		47.53 (4.82)	46.40 (5.04)	-2.57	0.011	0.229

AQ-27 = Attribution Questionnaire; MAKS = Mental Health Knowledge Schedule; RIBS = Reported and Intended Behaviour Scale; SD = standard deviation.

Data expressed as mean ± standard deviation, t test for independent samples. Effect size calculated with Cohen's *d*.

University students scored higher on the MAKS compared with the general population (p = 0.011; *d* = 0.229), suggesting greater knowledge of mental illness. Additionally, significant between-group differences were found in some MAKS items (supplementary Table S1). Specifically, a higher percentage of university students stated that psychotherapy can be an effective treatment for people with mental health problems (p = 0.009). Regarding the items on knowledge about various conditions, a higher proportion of university students agreed with the fact that schizophrenia (p = 0.005) and drug addiction (p = 0.008) were a mental illness. Conversely, a higher proportion of the general population stated that grief was a mental illness, compared with university students (p < 0.013).

Groups did not differ in total RIBS scores (Table 2). In addition, no significant differences were found between groups in the reported behavior items of the RIBS (p > 0.05 in all cases) (supplementary Table S2).

For the general population, higher scores for the AQ-27 coercion factor (p = 0.005, η² = 0.040) were observed among participants with an educational level of vocational training, compared to those with a university degree, with a small effect size (supplementary Table S3). For the total sample, higher scores for the AQ-27 coercion factor (p = 0.006, *d* = 0.251) were observed among participants with marital status of in a couple (including married), compared to those classed as single (including separated or divorced and widowed), with small effect sizes (supplementary Table S4). No significant differences were found in the scores for the AQ-27 factors according to personal history of mental illness diagnosis (p > 0.05) (supplementary Table S5).

### Relationships between stigmatizing attitudes, reported and intended behaviors, and previous contact with people with mental health problems and variables of interest

Total AQ-27, RIBS, and MAKS scores in both groups were analyzed according to sex (Table 3). Among university students, higher scores for the AQ-27 help factor (p = 0.002; *d* = 0.373) and lower scores for the segregation factor (p = 0.019; *d* = 0.324) were observed in women, with small effect sizes, when compared to men. Additionally, a trend to higher scores for the avoidance factor of the AQ-27 (p = 0.070; *d* = 0.242) was found in women.

**Table 3:** Total AQ-27, RIBS, and MAKS scores by sex in university students and the general population

		University students					General population				
Variables		Women (n = 138) Mean (SD)	Men (n = 87) Mean (SD)	*t* test	p-value	Cohen's *d*	Women (n = 194) Mean (SD)	Men (n = 85) Mean (SD)	*t* test	p-value	Cohen's *d*
AQ-27											
	Blame	8.27 (3.67)	9.16 (4.22)	-1.675	0.095	0.243	8.12 (3.47)	8.31 (3.48)	-0.415	0.679	0.051
	Pity	17.68 (4.59)	17.03 (4.7)	1.019	0.309	0.140	18.01 (4.68)	16.22 (4.31)	2.993	0.003	0.385
	Anger	7 (4.02)	7.48 (4.05)	-0.874	0.383	0.122	7.51 (3.83)	7.19 (3.97)	0.630	0.530	0.080
	Dangerousness	9.54 (5.61)	10.72 (5.42)	-1.557	0.121	0.215	10.40 (5.24)	10.32 (5.92)	0.119	0.905	0.015
	Fear	7.75 (5.74)	7.84 (5.3)	-0.112	0.911	0.016	8.73 (5.29)	7.60 (5.46)	1.600	0.111	0.205
	Help	23.72 (3.61)	22.17 (3.82)	3.072	0.002	0.373	22.68 (4.48)	21.44 (4.11)	2.182	0.030	0.298
	Coercion	17.63 (5.75)	18.59 (5.04)	-1.273	0.204	0.184	18.98 (5.18)	18.81 (4.34)	0.269	0.788	0.033
	Segregation	7.57 (4.77)	9.14 (4.98)	-2.356	0.019	0.324	7.95 (4.50)	8.47 (5.39)	-0.782	0.436	0.108
	Avoidance	11.33 (5.78)	12.75 (5.65)	-1.818	0.70	0.248	12.90 (6.11)	11.59 (5.68)	1.688	0.250	0.222
											
Total RIBS		15.81 (3.47)	15.14 (3.74)	1.377	0.170	0.191	14.92 (3.35)	15.47 (3.72)	-1.215	0.226	0.155
Total MAKS		47.44 (5.02)	47.64 (4.53)	-0.305	0.761	0.042	46.72 (5.06)	45.58 (4.91)	1.756	0.080	0.229

AQ-27 = Attribution Questionnaire; MAKS = Mental Health Knowledge Schedule; RIBS = Reported and Intended Behaviour Scale; SD = standard deviation.

Data expressed as mean ± standard deviation, t test for independent samples. Effect size calculated with Cohen's *d*.

Similarly, within the general population, women exhibited higher scores for the AQ-27 pity (p = 0.003; *d* = 0.385) and help factors (p = 0.030; *d* = 0.298) and a higher total score for the MAKS (p = 0.022; *d* = 0.307), when compared to men (Table 3).

Both groups’ scores for the AQ-27 factors were examined based on the reported behavior items of the RIBS. Among university students, those who had lived with someone with mental health problems had lower scores for the AQ-27 dangerousness (p = 0.025; *d* = 0.321), segregation (p = 0.041; *d* = 0.292), and fear factors (p = 0.042; *d* = 0.291), with small effect sizes, compared to those who had not lived with individuals with mental health problems. Those who had worked with someone with a mental health problem exhibited lower scores for the pity (p = 0.008; *d* = 0.389), anger (p = 0.043; *d* = 0.294), dangerousness (p < 0.001; *d* = 0.543), fear (p < 0.001; *d* = 0.518), and avoidance factors (p = 0.013; *d* = 0.364). Those who had had a close friend with a mental health problem had higher scores for the help factor (p = 0.005; *d* = 0.417). No significant differences were observed between university students who had had a neighbor with a mental health problem and those who had not (supplementary Table S6).

Conversely, in the general population, those who had lived with someone with mental health problems exhibited lower scores for the AQ-27 anger (p = 0.025; *d* = 0.288) and segregation factors (p = 0.048; *d* = 0.254). Additionally, they had higher scores for the help (p = 0.001; *d* = 0.407) factor with small effect sizes compared to those who had not lived with individuals with mental health problems. Those who had worked with someone with a mental health problem exhibited lower scores for the pity (p = 0.011; *d* = 0.335), anger (p = 0.004; *d* = 0.377), dangerousness (p = 0.003; *d* = 0.389), fear (p < 0.001; *d* = 0.467), segregation (p = 0.002; *d* = 0.415), and avoidance factors (p = 0.005; *d* = 0.370). Those who had had a neighbor with a mental health problem exhibited lower scores for the anger (p = 0.018; *d* = 0.335), dangerousness (p = 0.045; *d* = 0.287), and segregation factors (p = 0.011; *d* = 0.365). Those who had had a close friend with a mental health problem had lower scores for the anger (p = 0.009; *d* = 0.348), dangerousness (p = 0.004; *d* = 0.394), fear (p = 0.014; *d* = 0.331), and coercion factors (p = 0.033; *d* = 0.295) (supplementary Table S7).

To control the effect of several covariates in the differences between the two groups in social stigma as measured with the AQ-27 factors (dependent variable), a regression model was constructed including group (university students or the general population), age, and knowledge about mental illness (MAKS total score) (Table 4). University students displayed higher levels in the blame factor compared to the general population (p = 0.014). In other words, the two groups showed similar levels of social stigma and only differed in one AQ-27 factor, which in addition did not survive a multiple-comparison correction (p = 0.126). Age emerged as a significant independent predictor of all AQ-27 factors (p < 0.05 in all cases), indicating that older participants exhibited higher levels of stigmatizing attitudes. Additionally, the MAKS total score was negatively associated with the dangerousness (p = 0.002), fear (p = 0.008), avoidance (p = 0.025), and segregation factors (p = 0.037) and positively associated with the help factor (p = 0.013). This suggests that individuals with greater knowledge about mental health hold fewer stigmatizing attitudes.

**Table 4:** Regression models: contribution of different variables to each AQ-27 factor score

	Dependent variable								
	Blame	Pity	Anger	Dangerousness	Fear	Help	Coercion	Segregation	Avoidance
**Predictor**									
Constant	B = 6.087 (SE = 1.697), t = 3.587, p < 0.001	B = 15.451 (SE = 2.153), t = 7.177, p < 0.001	B = 8.520 (SE = 1.830), t = 4.656, p < 0.001	B = 15.083 (SE = 2.513), t = 6.002, p < 0.001	B = 11.648 (SE = 2.510), t = 4.640, p < 0.001	B = 19.275 (SE = 1.915), t = 10.064, p < 0.001	B = 15.640 (SE = 2.362), t = 6.621, p < 0.001	B = 10.134 (SE = 2.223), t = 4.560, p < 0.001	B = 14.116 (SE = 2.679), t = 5.269, p < 0.001
Age	B = 0.042 (SE = 0.13), t = 3.137, p = 0.002	B = 0.039 (SE = 0.017), t = 2.310, p = 0.021	B = 0.031 (SE = 0.014), t = 2.130, p = 0.034	B = 0.055 (SE = 0.020), t = 2.804, p = 0.005	B = 0.067 (SE = 0.020), t = 3.382, p = 0.001	B = −0.031 (SE = 0.015), t = −2.068, p = 0.039	B = 0.091 (SE = 0.019), t = 4.924, p < 0.001	B = 0.052 (SE = 0.017), t = 2.951, p = 0.003	B = 0.090 (SE = 0.021), t = 4.296, p > 0.001
Group (reference: general population)	B = 0.875 (SE = 0.356), t = 2.461, p = 0.014	B = 0.389 (SE = 0.451), t = 0.861, p = 0.390	B = 0.198 (SE = 0.384), t = 0.517, p = 0.605	B = 0.392 (SE = 0.527), t = 0.744, p = 0.457	B = 0.298 (SE = 0.526), t = 0.566, p = 0.572	B = 0.385 (SE = 0.401), t = 0.958, p = 0.338	B = 0.080 (SE = 0.495), t = 0.163, p = 0.871	B = 0.710 (SE = 0.466), t = 1.525, p = 0.128	B = 0.488 (SE = 0.561), t = 0.869, p = 0.385
Total MAKS	B = 0.007 (SE = 0.033), t = 0.222, p = 0.824	B = 0.007 (SE = 0.042), t = 0.179, p = 0.858	B = −0.052 (SE = 0.036), t = −1.455, p = 0.146	B = −0.152 (SE = 0.049), t = −3.110, p = 0.002	B = −0.131 (SE = 0.049), t = −2.684, p = 0.008	B = 0.093 (SE = 0.037), t = 2.491, p = 0.013	B = −0.012 (SE = 0.046), t = −0.268, p = 0.789	B = - 0.091 (SE = 0.043), t = −2.095, p = 0.037	B = −0.117 (SE = 0.052), t = −2.243, p = 0.025
Model summary	F(3,502) = 3.889, p = 0.009, R^2^adj = 0.017	F(3,502) = 1.780, p = 0.150, R^2^adj = 0.005	F(3,502) = 2.545, p = 0.055, R^2^adj = 0.009	F(3,502) = 6.692, p < 0.001, R^2^adj = 0.033	F(3,502) = 7.346, p < 0.001, R^2^adj = 0.036	F(3,502) = 5.542, p = 0.001, R^2^adj = 0.026	F(3,502) = 9.605, p < 0.001, R^2^adj = 0.049	F(3,502) = 4.838, p = 0.002, R^2^adj = 0.022	F(3,502) = 9.026, p < 0.001, R^2^adj = 0.046

AQ-27 = Attribution Questionnaire; B = coefficient estimates; F = F-statistic; MAKS = Mental Health Knowledge Schedule; R^2^adj = adjusted R^2^; SE = standard error; t = t-value.

Multivariate regression model applied.

## Discussion

The novelty of this study lies in its comprehensive evaluation of stigmatizing attitudes, reported and intended behavior towards mental illness, and knowledge of mental health among university students and the comparison with the general population.

Regarding the main objective, during the initial analysis, university students endorsed more positive attitudes, specifically in the help factor of the AQ-27, and knowledge about mental health compared to the general population. However, this difference in the help factor did not persist after adjusting for covariates in the regression model. After adjusting for age and knowledge about mental health in the regression model, students exhibited more negative attitudes than the general population in one specific AQ-27 factor, i.e., blame. Contrary to our hypothesis, overall, attitudes were more similar than different across both groups. The results post-adjustment suggest that age and knowledge about mental health play a crucial role in explaining the observed differences. Hence, the observed patterns in the initial analysis were likely influenced by the lower mean age and greater knowledge about mental health among university students compared to the general population. Notably, age emerged as a significant predictor across all AQ-27 factors in the regression model, revealing that older participants exhibited higher levels of stigmatizing attitudes. Similar comparative studies conducted in Arab-Islamic societies have not yielded conclusive findings.^24,25^ A study conducted in Oman found comparable attitudes towards individuals with mental illness among medical students, relatives of psychiatric patients, and the general population.^24^ Younger participants in this study showed a trend towards more favorable attitudes, although these differences were not statistically significant. On the other hand, another study in Pakistan revealed a noteworthy disparity in the social distance to mental illness among university students and the general population. The general population exhibited a significantly higher level of stigma, assessed through social distancing, in comparison to both healthcare students and professionals.^25^ After adjusting for age, gender, education, and profession, being over the age of 30 years was associated with higher levels of stigmatizing attitudes in Pakistan. It is noteworthy that, in contrast to our study, neither of the two previous studies controlled for the effect on stigmatizing attitudes of knowledge about mental illness. Within the regression model in our study, mental health knowledge was associated with four out of the nine AQ-27 factors. This suggests that individuals with a more comprehensive understanding of mental health exhibit fewer stigmatizing attitudes. Several studies have shown that a lack of education and knowledge about mental health is associated with higher levels of mental illness stigma among the general population and may perpetuate stereotypes and misconceptions.^36,37^ Thus, further investigations comparing these same two groups in Western countries are needed to deepen our understanding of how age and knowledge about mental illness influence social stigma.

Regarding the relationship between social stigma and other variables of interest, women from both study groups exhibited lower levels of stigmatizing attitudes than men. More specifically, among university students, women demonstrated a greater willingness to support individuals with mental illness and held fewer beliefs that people with mental illness should be excluded from society. Similarly, within the general population, women reported increased sympathy and a greater readiness to aid individuals with mental illness, coupled with greater knowledge about mental health compared to men. Although some studies have suggested that sex has no significant effect on attitudes, the sex differences identified in our study align with those reported in studies involving university students and the general population.^7,8,11,24,31,38^ A systematic review of population studies revealed that women tend to perceive individuals with mental disorders as less responsible for their illness and are more willing to volunteer and engage in the care of people with mental illness than men.^39^ This trend is influenced by complex interplays of societal expectations, communication styles, cultural norms, education initiatives, personal experiences, and mental health literacy.^7,8,11,24,31,38^

Individuals from both groups who had prior contact with someone with mental health issues – whether through living with, having a neighbor or friend, or working with people with mental health problems – showed lower levels of stigmatizing attitudes compared to those who had not. These results converge with those of other studies conducted in university students and the general population separately.^11,38,40–43^ Previous studies included in a recent systematic review support the theory that prior contact with individuals with mental illness is associated with reduced stigma.^44^ This is likely because direct exposure fosters understanding, empathy, and a more nuanced perspective, challenging preconceived notions and reducing the tendency to stigmatize individuals with mental health problems.^13,17,18,24^

The present findings further support the theory that higher levels of education and prior contact with mental illness are linked to decreased stigma. The Lancet Commission on ending stigma and discrimination in mental health found that promoting social interaction between individuals with and without personal experience of mental health conditions is the most effective, evidence-based approach to reducing stigmatization.^45^

In the literature, there are numerous examples of interventions conducted in university students and the general population intending to reduce the stigma towards people with mental illness. According to a recent systematic review and meta-analysis,^46^ contact-based and educational interventions can reduce the social stigma towards mental illness. On the one hand, contact interventions include exposure to people with SMI, both direct (in person) and indirect (via video), and are believed to work through anxiety reduction and increased empathy.^22^ On the other hand, educational interventions aim to reduce the social stigma against mental illness by providing information that contradicts society's stereotypes about this group. A meta-analysis that analyzed 72 articles from 12 countries found that contact interventions were more effective for adults, while educational strategies were more effective for adolescents.^47^ Moreover, the effect of contact interventions is significantly smaller on community members compared to university students.^48^

The interpretation of the present findings must be understood in the context of several limitations. First, causality cannot be established from a cross-sectional, observational study. Second, data collection through a sample survey may be biased by social desirability.^49^ Third, using a convenience sample and self-administered instruments are potential limitations of this study. However, many similar studies have previously used the same methodology, which suggests that the findings of this study may still be valid.^11–13,20,50,51^ In addition, the total response rate exceeded the minimum expected sample size (see methodology section), which can be considered a study strength. Furthermore, the two study groups had similar sociodemographic characteristics, all of which may increase the reliability of the present findings. Of note, this is among the few studies to characterize, in a comprehensive fashion, stigmatizing attitudes, previous contact with people with mental illness, and knowledge of mental health among university students, using the general population as a comparison group. Moreover, it is the first comparative study of its kind conducted in a Western society.

## Conclusion

Contrary to our hypothesis, university students exhibited stigmatizing attitudes at levels similar to those of the general population. The present findings suggest that, among both university students and the general population, female sex, older age, previous contact with individuals with mental illness, and greater knowledge of mental health are all associated with less stigma toward people with mental illness. Tailored interventions grounded in exposure to mental illness have the potential to help reduce stigmatizing attitudes within both groups. The results of this study are expected to establish a starting point for future research in this context. Further studies are essential to comprehensively analyze the effectiveness of diverse interventions aimed at reducing the prevailing stigma towards mental health within both university and general populations. Such research is vital to promote the social integration of individuals with mental health problems.

## References

[1] Corrigan PW (2000). Mental health stigma as social attribution: implications for research methods and attitude change. Clin Psychol (New York).

[2] Angermeyer MC, Matschinger H (2003). The stigma of mental illness: effects of labelling on public attitudes towards people with mental disorder. Acta Psychiatr Scand.

[3] Corrigan PW, Watson AC, Ottati V (2003). From whence comes mental illness stigma?. Int J Soc Psychiatry.

[4] Hinshaw SP, Cicchetti D (2000). Stigma and mental disorder: conceptions of illness, public attitudes, personal disclosure, and social policy. Dev Psychopathol.

[5] Brouwers EPM, Mathijssen J, Van Bortel T, Knifton L, Wahlbeck K, Van Audenhove C (2016). Discrimination in the workplace, reported by people with major depressive disorder: a cross-sectional study in 35 countries. BMJ Open.

[6] Organisation for Economic Co-operation and Development (OECD) (2012). Myths and realities about mental health and work.

[7] Mann CE, Himelein MJ (2004). Factors associated with stigmatization of persons with mental illness. Psychiatr Serv.

[8] Latalova K, Kamaradova D, Prasko J (2014). Perspectives on perceived stigma and self-stigma in adult male patients with depression. Neuropsychiatr Dis Treat.

[9] Boyd JE, Adler EP, Otilingam PG, Peters T (2014). Internalized Stigma of Mental Illness (ISMI) scale: a multinational review. Compr Psychiatry.

[10] Carrara BS, Ventura CAA, Bobbili SJ, Jacobina OMP, Khenti A, Mendes IAC (2019). Stigma in health professionals towards people with mental illness: an integrative review. Arch Psychiatr Nurs.

[11] Ruiz JC, Fuentes-Durá I, López-Gilberte M, Dasí C, Pardo-García C, Fuentes-Durán MC (2022). Public stigma profile toward mental disorders across different university degrees in the University of Valencia (Spain). Front Psychiatry.

[12] Shehata WM, Abdeldaim DE (2020). Stigma towards mental illness among Tanta University students, Egypt. Community Ment Health J.

[13] Lyndon AE, Crowe A, Wuensch KL, McCammon SL, Davis KB (2019). College students’ stigmatization of people with mental illness: familiarity, implicit person theory, and attribution. J Ment Health.

[14] Failde I, Salazar A, Elorza J, Casais L, Pérez V, Martínez LC (2014). Spanish medical students’ attitudes and views towards mental health and psychiatry: a multicentric cross-sectional study. Acad Psychiatry.

[15] Zabaleta R, Muñoz R, Martínez-Pérez A, Lezcano-Barbero F (2023). Mental illness stigma and attitudes among university students: descriptive study. Salud Drogas.

[16] Choe C, Baldwin ML, Song H (2020). A hierarchy of stigma associated with mental disorders. J Ment Health Policy Econ.

[17] Feeg VD, Prager LS, Moylan LB, Smith KM, Cullinan M (2014). Predictors of mental illness stigma and attitudes among college students: using vignettes from a campus common reading program. Issues Ment Health Nurs.

[18] Sandhu HS, Arora A, Brasch J, Streiner DL (2019). Mental health stigma: explicit and implicit attitudes of Canadian undergraduate students, medical school students, and psychiatrists. Can J Psychiatry.

[19] Yamaguchi S, Wu SI, Biswas M, Yate M, Aoki Y, Barley EA (2013). Effects of short-term interventions to reduce mental health-related stigma in university or college students: a systematic review. J Nerv Ment Dis.

[20] Chan JYN, Mak WWS, Law LSC (2009). Combining education and video-based contact to reduce stigma of mental illness: "The Same or Not the Same" anti-stigma program for secondary schools in Hong Kong. Soc Sci Med.

[21] Na JJ, Park JL, Khagva TL, Mikami AY (2022). The efficacy of interventions on cognitive, behavioral, and affective public stigma around mental illness: a systematic meta-analytic review. Stigma Health.

[22] Lien YY, Lin HS, Lien YJ, Tsai CH, Wu TT, Li H (2020). Challenging mental illness stigma in healthcare professionals and students: a systematic review and network meta-analysis. Psychol Health.

[23] Holzinger A, Kaup B, Gutiérrez-Lobos K (2002). Potentially dangerous behavior in the mentally ill: attitudes of journalists and medical students toward compulsory admission. Int J Offender Ther Comp Criminol.

[24] Al-Adawi S, Dorvlo ASS, Al-Ismaily SS, Al-Ghafry DA, Al-Noobi BZ, Al-Salmi A (2002). Perception of and attitude towards mental illness in Oman. Int J Soc Psychiatry.

[25] Husain MO, Zehra SS, Umer M, Kiran T, Husain M, Soomro M (2020). Stigma toward mental and physical illness: attitudes of healthcare professionals, healthcare students and the general public in Pakistan. BJPsych Open.

[26] Atienza-Carbonell B, Guillén V, Irigoyen-Otiñano M, Balanzá-Martínez V (2022). Screening of substance use and mental health problems among Spanish medical students: a multicenter study. J Affect Disord.

[27] Atienza-Carbonell B, Balanzá-Martínez V (2020). Prevalence of depressive symptoms and suicidal ideation among Spanish medical students. Actas Esp Psiquiatr.

[28] Atienza-Carbonell B, Hernández-Évole H, Balanzá-Martínez V (2022). A "patient as educator" intervention: reducing stigmatizing attitudes toward mental illness among medical students. Front Public Health.

[29] Johnson TP (2014). Snowball Sampling: Introduction.

[30] Masedo A, Grandón P, Saldivia S, Vielma-Aguilera A, Castro-Alzate ES, Bustos C (2021). A multicentric study on stigma towards people with mental illness in health sciences students. BMC Med Educ.

[31] Gallego J, Cangas AJ, Aguilar JM, Trigueros R, Navarro N, Galván B (2020). Education students’ stigma toward mental health problems: a cross-cultural comparison. Front Psychiatry.

[32] Corrigan P, Markowitz FE, Watson A, Rowan D, Kubiak MA (2003). An attribution model of public discrimination towards persons with mental illness. J Health Soc Behav.

[33] Muñoz M, Guillén AI, Pérez-Santos E, Corrigan PW (2015). A structural equation modeling study of the Spanish mental illness stigma attribution questionnaire (AQ-27-E). Am J Orthopsychiatry.

[34] Evans-Lacko S, Rose D, Little K, Flach C, Rhydderch D, Henderson C (2011). Development and psychometric properties of the Reported and Intended Behaviour Scale (RIBS): a stigma-related behaviour measure. Epidemiol Psychiatr Sci.

[35] Evans-Lacko S, Little K, Meltzer H, Rose D, Rhydderch D, Henderson C (2010). Development and psychometric properties of the mental health knowledge schedule. Can J Psychiatry.

[36] Yin H, Wardenaar KJ, Xu G, Tian H, Schoevers RA (2020). Mental health stigma and mental health knowledge in Chinese population: a cross-sectional study. BMC Psychiatry.

[37] Faruk MO, Khan AH, Chowdhury KUA, Jahan S, Sarker DC, Colucci E (2023). Mental illness stigma in Bangladesh: findings from a cross-sectional survey. Glob Ment Health (Camb)].

[38] Sandhu HS, Arora A, Brasch J (2019). Correlates of explicit and implicit stigmatizing attitudes of Canadian undergraduate university students toward mental illness: a cross-sectional study. J Am Coll Health.

[39] Holzinger A, Floris F, Schomerus G, Carta MG, Angermeyer MC (2012). Gender differences in public beliefs and attitudes about mental disorder in western countries: a systematic review of population studies. Epidemiol Psychiatr Sci.

[40] Adewuya AO, Makanjuola ROA (2005). Social distance towards people with mental illness amongst Nigerian university students. Soc Psychiatry Psychiatr Epidemiol.

[41] Anosike C, Ukwe CV, Oparah AC (2020). Attitudes of pharmacy and non-pharmacy students towards mental illness in Nigeria: a comparative survey. Int J Pharm Pract.

[42] Chung KF, Chen EYH, Liu CSM (2001). University students’ attitudes towards mental patients and psychiatric treatment. Int J Soc Psychiatry.

[43] Vezzoli R, Archiati L, Buizza C, Pasqualetti P, Rossi G, Pioli R (2001). Attitude towards psychiatric patients: a pilot study in a northern Italian town. Eur Psychiatry.

[44] Zamorano S, Sáez-Alonso M, González-Sanguino C, Muñoz M (2023). Social stigma towards mental health problems in Spain: a systematic review. Clin Salud.

[45] Thornicroft G, Sunkel C, Alikhon Aliev A, Baker S, Brohan E, el Chammay R (2022). The Lancet Commission on ending stigma and discrimination in mental health. Lancet.

[46] Morgan AJ, Reavley NJ, Ross A, Too LS, Jorm AF (2018). Interventions to reduce stigma towards people with severe mental illness: systematic review and meta-analysis. J Psychiatr Res.

[47] Corrigan PW, Morris SB, Michaels PJ, Rafacz JD, Rüsch N (2012). Challenging the public stigma of mental illness: A meta-analysis of outcome studies. Psychiatric Services.

[48] Maunder RD, White FA (2019). Intergroup contact and mental health stigma: a comparative effectiveness meta-analysis. Clin Psychol Rev.

[49] Althubaiti A (2016). Information bias in health research: definition, pitfalls, and adjustment methods. J Multidiscip Healthc.

[50] Oliveira AM, Machado D, Fonseca JB, Palha F, Silva Moreira P, Sousa N (2020). Stigmatizing attitudes toward patients with psychiatric disorders among medical students and professionals. Front Psychiatry.

[51] Babicki M, Małecka M, Kowalski K, Bogudzińska B, Piotrowski P (2021). Stigma levels toward psychiatric patients among medical students—a worldwide online survey across 65 countries. Front Psychiatry.

