# Supplementary material for: Stigmatizing attitudes towards mental illness among university students: a comparative study with the general population
Source: Trends Psychiatry Psychother. 2024 Oct 28;46:e20230708. doi: 10.47626/2237-6089-2023-0708 (PMC11565248; doi:10.47626/2237-6089-2023-0708)
Supplement: Supplementary file 1 [file 2238-0019-trends-46-e20230708-suppl1.pdf]

http://doi.org/10.47626/2237-6089-2023-0708

Table S1 - Results from the MAKS reported items by group

MAKS **items**	**University students (n = 226) n (%)**	**General population (n = 280) n (%)**	**X²**	**p-value**
Most people with mental health problems want to have paid employment.	156 (69.0)	176 (62.9)	2.110	0.146
If a friend had a mental health problem. I know what advice to give them to get professional help.	158 (69.9)	177 (63.2)	2.507	0.113
Medication can be an effective treatment for people with mental health problems.	198 (87.6)	228 (81.4)	3.591	0.058
Psychotherapy (e.g., talking therapy or counselling) can be an effective treatment for people with mental health problems.	195 (86.3)	216 (77.1)	6.851	0.009
People with severe mental health problems can fully recover.	94 (41.6)	97 (34.6)	2.571	0.109
Most people with mental health problems go to a healthcare professional to get help.	56 (24.8)	79 (28.2)	0.755	0.385
Depression*	185 (81.9)	231 (82.5)	0.035	0.851
Stress*	98 (43.4)	126 (45.0)	0.136	0.712
Schizophrenia*	222 (98.2)	260 (92.9)	7.991	0.005
Bipolar disorder (manic-depression)*	219 (96.9)	262 (93.6)	2.955	0.086
Drug addiction*	153 (67.7)	157 (56.1)	7.125	0.008
Grief*	65 (28.8)	110 (39.3)	6.123	0.013

MAKS = Mental Health Knowledge Schedule; X² = chi-square.
Data expressed as absolute frequency (%), X² test of association,
*Agreement that the condition is a type of mental illness.

http://doi.org/10.47626/2237-6089-2023-0708

Table S2 - Results for the RIBS reported items by group

RIBS items	University students (n = 226) n (%)	General population (n = 280) n (%)	X^2	p-value
Are you currently living with, or have you ever lived with someone with a mental health problem?	88 (38.9)	101 (36.1)	3.423	0.181
Are you currently working with, or have you ever worked with someone with a mental health problem?	100 (44.2)	121 (43.2)	0.406	0.816
Do you currently have, or have you ever had a neighbor with a mental health problem?	91 (40.3)	107 (38.2)	0.287	0.866
Do you currently have, or have you ever had a close friend with a mental health problem?	142 (62.8)	172 (61.4)	1.605	0.448

RIBS = Reported and Intended Behaviour Scale; X^2 = chi-square.
Data expressed as absolute frequency (%), X^2 test of association.

Table S3 - Comparison of total scores on the AQ-27 according to the educational level of the general population

Variables	Secondary education or lower (n = 26) Mean (SD)	Vocational training (n = 50) Mean (SD)	University degree (n = 188) Mean (SD)	F(2,261)	p-value	η^2
Blame	8.85 (4.57)	8.66 (3.62)	7.88 (3.18)	1.683	0.188	0.013
Pity	17.85 (5.04)	18.02 (5.07)	17.28 (4.45)	0.603	0.548	0.005
Anger	9.04 (4.64)	7.1 (3.89)	7.34 (3.76)	2.469	0.087	0.019
Dangerousness	11.15 (5.7)	10.28 (5.03)	10.49 (5.51)	0.228	0.796	0.002
Fear	9.73 (5.67)	8.3 (5.57)	8.35 (5.47)	0.749	0.474	0.006
Help	21.35 (4.87)	22.6 (4.77)	22.29 (4.36)	0.686	0.505	0.005
Coercion	20.73 (3.66)	20.46 (4.61)	18.38 (5.06)	5.447	0.005*	0.040
Segregation	9.46 (5.84)	8.6 (4.54)	7.97 (4.75)	1.270	0.283	0.010
Avoidance	14.27 (5.74)	12.1 (5.41)	12.54 (6.19)	1.180	0.309	0.009

AQ-27 = Attribution Questionnaire; F = F-statistic; SD = standard deviation; η^2 = eta squared.
*Post hoc comparisons: vocational training > university degree.

http://doi.org/10.47626/2237-6089-2023-0708

http://doi.org/10.47626/2237-6089-2023-0708

Table S4 - Comparison of total scores on the AQ-27 according to the marital status of the entire sample

Variables	Single* (N = 192) Mean (SD)	In couple† (N = 314) Mean (SD)	*t* test	p-value	Cohen's *d*
Blame	8.57 (3.78)	8.27 (3.61)	0.898	0.370	0.081
Pity	17.06 (4.29)	17.67 (4.82)	-1.435	0.152	0.134
Anger	7.42 (3.94)	7.26 (3.95)	0.435	0.141	0.040
Dangerousness	10.05 (5.18)	10.29 (5.67)	-0.490	0.625	0.044
Fear	7.76 (5.14)	8.35 (5.69)	-1.180	0.239	0.109
Help	22.26 (4.22)	22.87 (4.12)	-1.604	0.109	0.146
Coercion	17.71 (5.23)	19.01 (5.12)	-2.743	0.006	0.251
Segregation	7.92 (4.43)	8.26 (5.05)	-0.779	0.436	0.071
Avoidance	11.89 (5.69)	12.45 (6)	-1.039	0.299	0.139

AQ-27 = Attribution Questionnaire; SD = standard deviation.
Data expressed as mean ± standard deviation, t test for independent samples. Effect size calculated with Cohen's *d*.
* Includes single, separated or divorced and widowed; † Includes in a couple and married.

Table S5 - Comparison of total scores on the AQ-27 according to the personal history of mental illness diagnosis of the entire sample

Variables	Previous diagnose (n = 77) Mean (SD)	No previous diagnose (n = 429) Mean (SD)	*t* test	p-value	Cohen's *d*
Blame	7.91 (3.18)	8.47 (3.73)	-1.236	0.217	0.161
Pity	17.32 (4.69)	17.45 (4.62)	-0.226	0.821	0.028
Anger	6.84 (3.50)	7.41 (4.02)	-1.160	0.247	0.151
Dangerousness	9.25 (4.72)	10.37 (5.60)	-1.659	0.098	0.216
Fear	7.40 (5.04)	8.26 (5.56)	-1.261	0.208	0.162
Help	23.60 (3.49)	22.46 (4.26)	2.212	0.027	0.293
Coercion	18.00 (5.41)	18.61 (5.16)	-0.942	0.347	0.115
Segregation	7.17 (3.92)	8.30 (4.95)	-1.904	0.057	0.253
Avoidance	11.56 (5.69)	12.35 (5.92)	-1.093	0.275	0.136

AQ-27 = Attribution Questionnaire; SD = standard deviation.
Data expressed as mean ± standard deviation, t test for independent samples. Effect size calculated with Cohen's *d*.

http://doi.org/10.47626/2237-6089-2023-0708

Table S6 - Comparison of total scores on the AQ-27 according to the reported behavior items of the RIBS among university students

	AQ-27								
	**Blame Mean (SD)**	**Pity Mean (SD)**	**Anger Mean (SD)**	**Dangerousness Mean (SD)**	**Fear Mean (SD)**	**Help Mean (SD)**	**Coercion Mean (SD)**	**Segregation Mean (SD)**	**Avoidance Mean (SD)**
RIBS-lived									
Yes (n = 88)	8.16 (3.77)	16.83 (4.68)	6.82 (3.83)	8.97 (5.3)	6.8 (5.13)	23.32 (4.04)	17.26 (6.14)	7.38 (4.54)	11.13 (6.03)
No (n = 116)	8.89 (3.95)	17.84 (4.72)	7.41 (4.04)	10.71 (5.54)	8.35 (5.57)	22.87 (3.51)	18.53 (4.74)	8.77 (4.98)	12.47 (5.45)
	t = -1.331, p = 0.185, *d* = 0.189	t = -1.514, p = 0.132, *d* = 0.214	t = -1.050, p = 0.295, *d* = 0.150	t = -2.265. p = 0.025, *d* = 0.321	t = -2.048, p = 0.042, *d* = 0.291	t = 0.844, p = 0.399, *d* = 0.118	t = -1.671, p = 0.096, *d* = 0.232	t = -2.055, p = 0.041, *d* = 0.292	t = -1.672, p = 0.096, *d* = 0.235
RIBS-worked									
Yes (n = 100)	8.56 (4.2)	16.52 (4.74)	6.65 (3.89)	8.42 (5.27)	6.38 (4.78)	23.3 (4.27)	17.34 (6.02)	7.72 (4.83)	10.86 (6.13)
No (n = 92)	8.8 (3.74)	18.33 (4.53)	7.85 (4.26)	11.38 (5.63)	9.21 (6.05)	22.72 (3.47)	18.82 (4.93)	8.86 (5.04)	12.93 (5.22)
	t = -0.424, p = 0.672, *d* = 0.061	t = -2.694, p = 0.008, *d* = 0.389	t = -2.036, p = 0.043, *d* = 0.294	t = -3.764, p < 0.001 *d* = 0.543	t = -3.606, p < 0.001 *d* = 0.518	t = 1.032, p = 0.303, *d* = 0.15	t = -1.848, p = 0.066, *d* = 0.268	t = -1.599, p = 0.111, *d* = 0.231	t = -2.512, p = 0.013, *d* = 0.364
RIBS-neighbor									
Yes (n = 91)	8.26 (3.76)	17.48 (4.95)	6.66 (3.77)	9.31 (5.56)	7.25 (5.61)	22.87 (4.09)	17.87 (5.59)	7.97 (4.73)	11.93 (5.95)
No (n = 73)	9.03 (3.67)	17.71 (4.59)	7.74 (4.42)	10.97 (5.56)	8.92 (5.82)	22.67 (3.81)	18.33 (5.13)	8.96 (5.26)	12.23 (5.55)
	t = -1.305, p = 0.194, *d* = 0.205	t = -0.304, p = 0.762, *d* = 0.048	t = -1.69, p = 0.093, *d* = 0.263	t = -1.907, p = 0.058, *d* = 0.300	t = -1.857, p = 0.065, *d* = 0.291	t = 0.316, p = 0.753, *d* = 0.050	t = -0.544, p = 0.587, *d* = 0.086	t = -1.269, p = 0.206, *d* = 0.198	t = -0.329, p = 0.742, *d* = 0.052
RIBS-friend									
Yes (n = 142)	8.35 (3.58)	17.55 (4.55)	7.15 (4.05)	9.72 (5.59)	7.77 (5.8)	23.61 (3.63)	18.11 (5.56)	8.02 (4.96)	11.7 (5.62)
No (n = 65)	9.35 (4.53)	17.48 (4.6)	7.45 (4.28)	10.74 (5.65)	8.22 (5.29)	22.03 (3.92)	18.49 (5.05)	8.91 (5.04)	12.42 (5.8)
	t = -1.726, p = 0.086, *d* = 0.247	t = 0.106, p = 0.916, *d* = 0.016	t = -0.472, p = 0.638, *d* = 0.007	t = -1.214, p = 0.226, *d* = 0.181	t = -0.521, p = 0.603, *d* = 0.079	t = 2.826, p = 0.005, *d* = 0.417	t = -0.469, p = 0.639, *d* = 0.071	t = -1.188, p = 0.236, *d* = 0.177	t = -0.845, p = 0.399, *d* = 0.126

AQ-27 = Attribution Questionnaire; RIBS = Reported and Intended Behaviour Scale.
Data expressed as mean ± standard deviation, t test for independent samples. Effect size calculated with Cohen's *d*.

http://doi.org/10.47626/2237-6089-2023-0708

Table S7 - Comparison of total scores on the AQ-27 according to the reported behavior items of the RIBS among the general population

	AQ-27								
	**Blame Mean (SD)**	**Pity Mean (SD)**	**Anger Mean (SD)**	**Dangerousness Mean (SD)**	**Fear Mean (SD)**	**Help Mean (SD)**	**Coercion Mean (SD)**	**Segregation Mean (SD)**	**Avoidance Mean (SD)**
RIBS-lived									
Yes (n = 101)	8.25 (3.45)	17.8 (4.74)	6.8 (3.66)	9.61 (5.43)	7.87 (5.82)	23.34 (3.6)	18.38 (5.23)	7.4 (4.56)	11.87 (5.84)
No (n = 162)	8.19 (3.62)	17.26 (4.58)	7.91 (4.01)	10.9 (5.44)	8.84 (5.25)	21.63 (4.71)	19.22 (4.8)	8.59 (4.86)	13.04 (6.02)
	t = 0.125, p = 0.901, *d* = 0.016	t = 0.923, p = 0.357, *d* = 0.54	t = -2.248, p = 0.025, *d* = 0.288	t = -1.868, p = 0.063, *d* = 0.237	t = -1.395, p = 0.164, *d* = 0.175	t = 3.119, p = 0.002, *d* = 0.407	t = -1.333, p = 0.184, *d* = 0.167	t = -1.988, p = 0.048, *d* = 0.254	t = -1.553, p = 0.122, *d* = 0.198
RIBS-worked									
Yes (n = 121)	7.99 (3.47)	16.86 (4.54)	6.74 (3.61)	9.14 (5.09)	6.98 (4.65)	22.64 (4.37)	18.36 (4.59)	7.14 (4.2)	11.42 (6.14)
No (n = 111)	8.48 (3.47)	18.42 (4.79)	8.2 (4.13)	11.22 (5.57)	9.43 (5.77)	22.28 (4.43)	19.61 (5.24)	9.1 (5.2)	13.63 (5.79)
	t = -1.065, p = 0.288, *d* = 0.140	t = -2.55, p = 0.011, *d* = 0.335	t = -2.877, p = 0.004, *d* = 0.377	t = -2.965, p = 0.003, *d* = 0.389	t = -3.572, p < 0.001, *d* = 0.467	t = 0.632, p = 0.528, *d* = 0.083	t = -1.949, p = 0.052, *d* = 0.255	t = -3.17, p = 0.002, *d* = 0.415	t = -2.813, p = 0.005, *d* = 0.370
RIBS-neighbor									
Yes (n = 107)	8.55 (3.17)	17.83 (4.85)	6.95 (3.18)	9.75 (5.37)	8.1 (5.66)	22.96 (3.86)	18.96 (4.75)	7.46 (4.57)	12.6 (6.16)
No (n = 91)	8.15 (3.62)	18.15 (4.86)	8.26 (4.52)	11.32 (5.59)	9.25 (5.75)	22.14 (4.59)	19.4 (5.52)	9.21 (5.01)	13.43 (6.1)
	t = 0.824, p = 0.411, *d* = 0.117	t = -0.465, p = 0.642, *d* = 0.066	t = -2.384, p = 0.018, *d* = 0.335	t = -2.013, p = 0.045, *d* = 0.287	t = -1.414, p = 0.159, *d* = 0.202	t = 1.365, p = 0.174, *d* = 0.193	t = -0.593, p = 0.554, *d* = 0.084	t = -2.569, p = 0.011, *d* = 0.365	t = -0.949, p = 0.344, *d* = 0.135
RIBS-friend									
Yes (n = 172)	8.05 (3.24)	17.54 (4.7)	6.94 (3.59)	9.62 (5.12)	7.8 (5.12)	22.8 (4.17)	18.51 (4.84)	7.71 (4.48)	12.27 (6.1)
No (n = 75)	8.25 (3.69)	17.53 (4.97)	8.33 (4.37)	11.81 (5.99)	9.64 (5.96)	22.19 (4.26)	19.96 (5.03)	8.72 (5.31)	12.05 (5.54)
	t = -0.43, p = 0.668, *d* = 0.058	t = 0.011, p = 0.991, *d* = 0.002	t = -2.616, p = 0.009, *d* = 0.348	t = -2.942, p = 0.004, *d* = 0.394	t = -2.466, p = 0.014, *d* = 0.331	t = 1.06, p = 0.290, *d* = 0.146	t = -2.146, p = 0.033, *d* = 0.295	t = -1.538, p = 0.125, *d* = 0.206	t = 0.261, p = 0.795, *d* = 0.037

AQ-27 = Attribution Questionnaire; RIBS = Reported and Intended Behaviour Scale; SD = standard deviation.
Data expressed as mean ± standard deviation, t test for independent samples. Effect size calculated with Cohen's *d*.
